# Neural Mechanisms of Neuro-Rehabilitation Using Transcranial Direct Current Stimulation (tDCS) over the Front-Polar Area

**DOI:** 10.3390/brainsci13111604

**Published:** 2023-11-18

**Authors:** Koji Ishikuro, Noriaki Hattori, Hironori Otomune, Kohta Furuya, Takeshi Nakada, Kenichiro Miyahara, Takashi Shibata, Kyo Noguchi, Satoshi Kuroda, Yuji Nakatsuji, Hisao Nishijo

**Affiliations:** 1 Department of Rehabilitation, Toyama University Hospital, Toyama 930-0194, Japan; ishikuro@med.u-toyama.ac.jp (K.I.); hattorin@med.u-toyama.ac.j (N.H.); hotomune@med.u-toyama.ac.jp (H.O.); furuyapt@med.u-toyama.ac.jp (K.F.); nakadapt@med.u-toyama.ac.jp (T.N.); 2 Department of Physical Therapy, Toyama College of Medical Welfare, Toyama 930-0194, Japan; miyahara@tif.ac.jp; 3 Department of Neurosurgery, Toyama Nishi General Hospital, Toyama 939-2716, Japan; sibata@dj8.so-net.ne.jp; 4 Department of Neurosurgery, Faculty of Medicine, University of Toyama, Toyama 930-0194, Japan; skuroda@med.u-toyama.ac.jp; 5 Department of Radiology, Faculty of Medicine, University of Toyama, Toyama 930-0194, Japan; kyo@med.u-toyama.ac.jp; 6 Department of Neurology, Faculty of Medicine, University of Toyama, Toyama 930-0194, Japan; nakatsuj@med.u-toyama.ac.jp; 7 Faculty of Human Sciences, University of East Asia, Shimonoseki 751-8503, Japan

**Keywords:** transcranial direct current stimulation, Parkinson’s disease, neural mechanisms, the frontal pole area

## Abstract

Transcranial direct current stimulation (tDCS) is a noninvasive brain stimulation (NIBS) technique that applies a weak current to the scalp to modulate neuronal excitability by stimulating the cerebral cortex. The technique can produce either somatic depolarization (anodal stimulation) or somatic hyperpolarization (cathodal stimulation), based on the polarity of the current used by noninvasively stimulating the cerebral cortex with a weak current from the scalp, making it a NIBS technique that can modulate neuronal excitability. Thus, tDCS has emerged as a hopeful clinical neuro-rehabilitation treatment strategy. This method has a broad range of potential uses in rehabilitation medicine for neurodegenerative diseases, including Parkinson’s disease (PD). The present paper reviews the efficacy of tDCS over the front-polar area (FPA) in healthy subjects, as well as patients with PD, where tDCS is mainly applied to the primary motor cortex (M1 area). Multiple evidence lines indicate that the FPA plays a part in motor learning. Furthermore, recent studies have reported that tDCS applied over the FPA can improve motor functions in both healthy adults and PD patients. We argue that the application of tDCS to the FPA promotes motor skill learning through its effects on the M1 area and midbrain dopamine neurons. Additionally, we will review other unique outcomes of tDCS over the FPA, such as effects on persistence and motivation, and discuss their underlying neural mechanisms. These findings support the claim that the FPA could emerge as a new key brain region for tDCS in neuro-rehabilitation.

## 1. Introduction

Techniques for noninvasive brain stimulation (NIBS) have been widely used in both patients and healthy people. A more accurate description of NIBS is that it is a low-intensity transcranial stimulation to modulate cortical excitability. There are two well-known neuromodulation therapies based on this strategy: repetitive transcranial magnetic stimulation (rTMS) and transcranial direct current stimulation (tDCS). rTMS applies magnetism to modulate cortical excitability, while tDCS uses electrical stimulation instead. While rTMS can stimulate the cortex with minimal reduction in stimulation intensity from the scalp, it is essential for the patient’s head to be secured to a comfortable chair to ensure precise stimulation of the region of interest (ROI). However, clinical situations can pose a challenge for rTMS, as patients may often exhibit involuntary movements that hinder fixation of the head position. Additionally, while it is infrequent, rTMS can cause harmful effects such as seizures. Conversely, tDCS has advantages in hospital settings, such as ease of usage, fewer adverse events, and high portability. Therefore, tDCS can be used in combination with physical rehabilitation to enhance plastic changes in ongoing synapses activated during physical rehabilitation [1,2]. Furthermore, recent research has consistently demonstrated that the combination of tDCS and rehabilitation is more effective than utilizing tDCS alone in the long-term improvement of neurological disease symptoms [1,2,3,4,5,6].

tDCS can safely modulate cortical activity in both acute (immediate) and after (chronic) effects. In acute effects during stimulation, tDCS can modulate cortical neuronal activity. For example, anodal stimulation can increase cortical excitability by depolarizing the soma and basal dendrite of cortical pyramidal neurons (somatic hypothesis) [7]. On the other hand, cathodal stimulation can produce opposite effects, i.e., hyperpolarization of the basal dendrites and soma [8,9]. Consistent with the somatic hypothesis, firing rates of putative pyramidal neurons were elevated by anodal stimulation and suppressed by cathodal stimulation in monkeys [10]. However, firing rates of putative inhibitory neurons increased with both anodal and cathodal stimulation (i.e., regardless of polarity). This is likely due to network-mediated effects resulting from the modulation of other cell types [10]. In after effects, tDCS may modulate cortical and subcortical synaptic connections (synaptic plasticity) [2]. Previous studies have reported that tDCS can facilitate motor learning through synaptic plasticity. This effect is mediated through the induction of nitric oxide (NO), activation of neurotrophic tyrosine kinase receptor type 2 (TrkB), and expression of brain-derived neurotrophic factor (BDNF) to promote synaptic plasticity [11,12]. These molecular mechanisms might underlie the molecular backgrounds for long-term potentiation (LTP) and long-term depression (LTD), and modulate synaptic connections in after effects. Therefore, due to its safety, application of tDCS to modulate cortical activity has been increasing in basic and clinical research since 2000 [13,14,15]. 

Thus, tDCS is increasingly being applied as a therapy for the motor symptoms of diverse brain disorders such as Parkinson’s disease (PD). Recently, the front-polar area (FPA) (Brodmann area 10) has garnered increased attention in motor learning, an important factor in rehabilitation. This paper aims to review the role of the FPA in motor learning and to review the current clinical efficacy of tDCS over various brain regions, including the FPA in PD. Our recent findings in healthy adults show that tDCS over the FPA enhances motor skill learning through its effects on the motor-related regions [16,17,18,19,20] (see below for details). We discuss how tDCS over the FPA may facilitate motor skill learning by affecting the motor-related regions. Consistent with these findings, we will show that tDCS over the FPA is also effective in PD. Another significant issue in rehabilitation is that a substantial proportion of patients discontinue rehabilitation programs due to a loss of persistence or motivation [17,18,19]. This paper also discusses the neural mechanisms of another effect of tDCS over the FPA, especially its effect on persistence and motivation. This motivational aspect is unique to tDCS over the FPA compared to tDCS over the motor-related regions. These findings suggest that the FPA could be a novel brain region of interest for tDCS in neuro-rehabilitation.

## 2. tDCS Effects of the Front-Polar Area (FPA) in Rehabilitation

### 2.1. A Role of the FPA in Motor Learning and Rehabilitation

**Figure 1:**
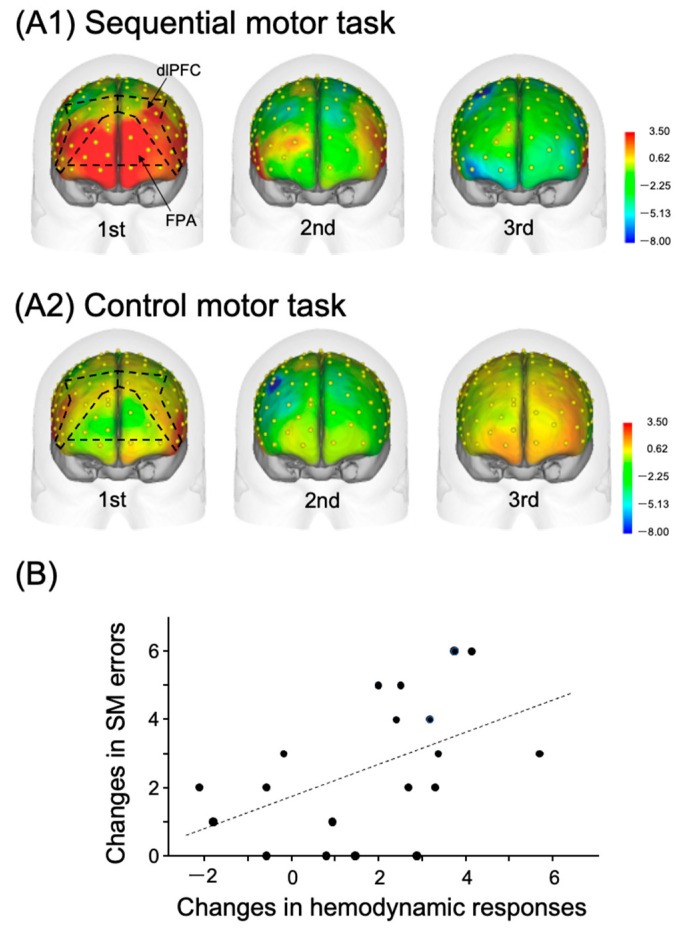
Relations between the PFC hemodynamic activity in the hand movement tasks and task performance. Reproduced from Kobayashi et al. (2021) [16] under a CC-BY license. Topographical maps of the PFC cortical activity shown as effect sizes in the three consecutive trials in the sequential motor (SM) (**A1**) and control motor (**A2**) tasks. NIRS recording channels on the head are shown as yellow dots. (**B**) Positive relationships between changes in PFC cortical activity and SM-task error reduction, which occurred on the first and second trials. The dotted line indicates a regression line.

Multiple lines of evidence support the notion that the FPA may represent a new therapeutic target for tDCS-based rehabilitation, aimed at motor learning. The role played by the most anterior portion of the prefrontal cortex (PFC) (i.e., FPA) in motor learning is unique in various regards. Some previous noninvasive imaging studies have demonstrated that the FPA is mainly activated when subjects acquire novel motor task(s) [21,22,23]. Recently, Kobayashi et al. (2021) examined FPA activity during the acquisition of a sequential motor task with near-infrared spectroscopy (NIRS) [16]. The study required inexperienced participants to sequentially lay the right hands on a table at a steady pace of three movements per sec, using three distinct hand postures: a fist held vertically, a palm held vertically, and a palm held horizontally [sequential motor (SM) task]. In the control motor task, the subjects repeatedly tapped the table with their right palm at the same speed. This control task could be executed without any motor learning. For both tasks, the participants completed three trials in 30 sec per trial, and performance errors were counted in each trial [16]. The results indicated that hemodynamic cortical activity (Oxy-Hb) was prominent in the PFC, including the FPA and dlPFC, in the first trial, but decreased in later trials in the SM task, as shown in Figure 1(A1). No such prominent responses were observed in the control motor task, as shown in Figure 1(A2). Cortical activity changes between the initial (first) and subsequent (second) trials in the sequential motor task, reflected in Figure 1B, were positively correlated with error reduction between the initial and subsequent trials, representing performance improvement by learning. These findings support the notion that the FPA contributes significantly to motor learning, as there is a positive relation between larger hemodynamic cortical activity changes in the PFC, including the FPA, and a larger performance improvement.

**Figure 2:**
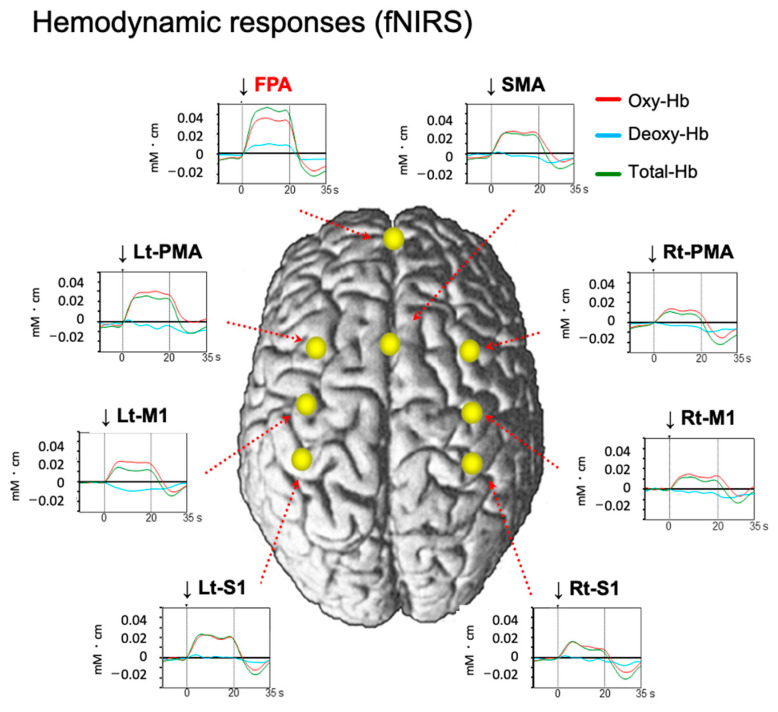
Averaged hemodynamic responses during a performance of a peg board task. Reproduced from Ishikuro et al. (2014) [17] under a CC-BY license. Cortical activity (changes in Oxy-Hb) rapidly increased during the task at the FPA and seven somatosensory and motor-related areas (supplementary motor area (SMA), left premotor area (Lt-PMA), right premotor area (Rt-PMA), left primary motor area (Lt-M1), right primary motor area (Rt-M1), left primary somatosensory area (Lt-S1), and right primary somatosensory area (Rt-S1)). Changes in Oxy-Hb, total-Hb, and deoxy-Hb concentrations are shown by red, green, and blue lines, respectively. Arrows represent the onset of the task.

**Figure 3:**
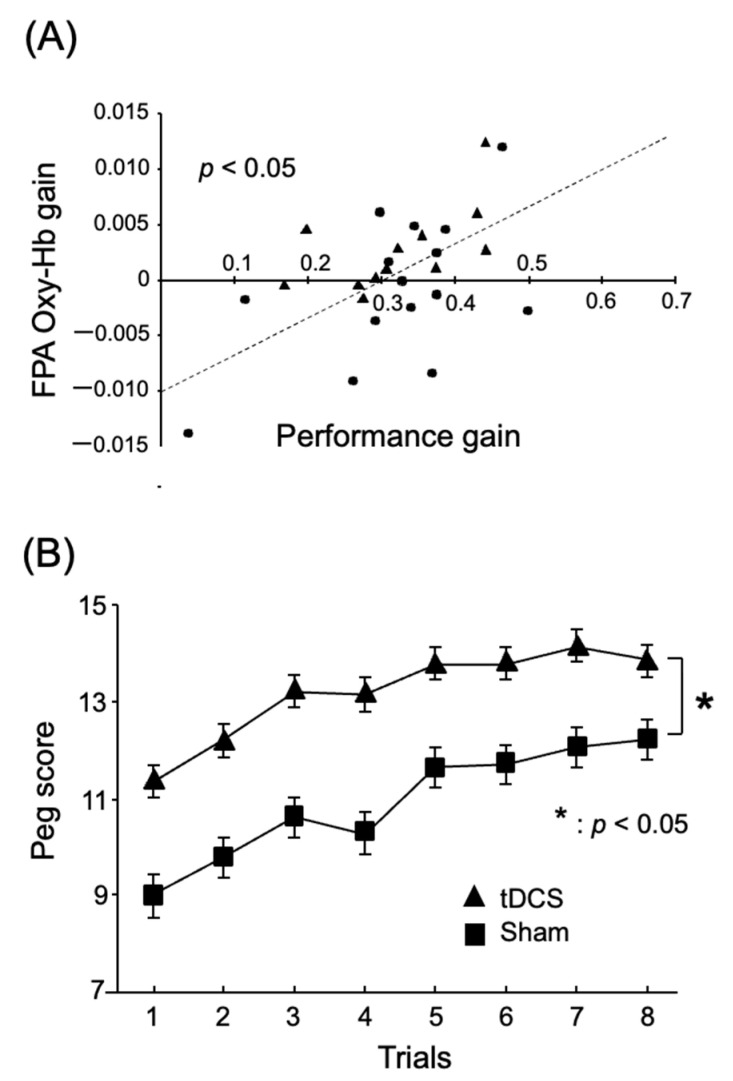
FPA role in motor learning in a peg board task. Reproduced from Ishikuro et al. (2014) [17] under a CC-BY license. (**A**) Positive relations between task performance gain and FPA Oxy-Hb gain. The dotted line indicates a regression line. Each filled symbol indicates data for each subject. (**B**) Effects of tDCS over the FPA on task performance (peg scores) in the peg board task. *, *p* < 0.05.

These results suggest that the FPA plays a role in motor rehabilitation, where motor learning plays a crucial part. Ishikuro et al. (2014) examined the function of the FPA in motor learning in rehabilitation using NIRS [17]. Healthy participants were asked to perform a peg board task for upper-extremity functions in the simple test for evaluating hand function (STEF): they picked up a peg using their right thumb and index finger and inserted it into a hole in the STEFF board. The pegs are small pins used for this activity. In each of the eight trials, the subjects had to repeat these actions as fast as possible for 20 seconds. It is noteworthy that the subjects were required to continuously improve their performance over the eight trials, which contrasts with the study of Kobayashi et al. (2021). The results again indicated that the FPA was highly active during this task in which continuous performance improvement was required (Figure 2). Furthermore, both behavioral performance (peg score, i.e., the number of pegs inserted into the peg holes per trial) and cortical activity in the FPA during the peg board task increased incrementally in subsequent trials. To measure the incremental speed of these two parameters across the eight trials in each subject, both parameters were subjected to simple linear regression analysis. “Incremental speed of hemodynamic activity (Oxy-Hb gain)” was assessed by the slope of this fitted line. In the same way, “Incremental speed of behavioral performance (performance gain)” was evaluated by its slope of the fitted line. Figure 3A illustrates that performance gain is positively associated with Oxy-Hb gain in the FPA. This suggests that the FPA helps to promote motor learning. To further test this possibility, Ishikuro et al. (2014) applied anodal tDCS over the FPA before the peg board task. As a result, the findings demonstrate that anodal tDCS increased peg scores, as shown in Figure 3B. Furthermore, Oxy-Hb gain in the FPA was positively associated with those in the left premotor area (Lt-PMA), left primary motor area (Lt-M1), and left primary somatosensory area (Lt-S1). This suggests that the FPA promotes motor learning through its effects over the somatosensory and motor-related areas.

### 2.2. Modulatory Effects of the FPA on the Motor-Related Regions

**Figure 4:**
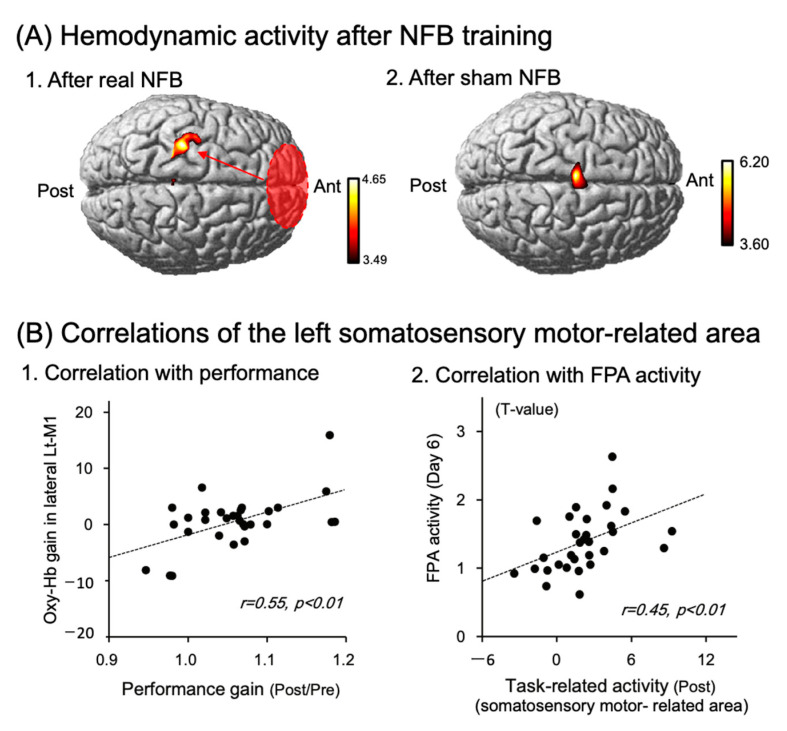
FPA role in improving performance in neurofeedback (NFB) training for six days. Reproduced from Ota et al. (2020) [20] under a CC-BY license. (**A**) Averaged task-related responses, shown as NIRS-SPM T-statistic maps, during a performance of a peg board task after the real (**A1**) and sham (**A2**) neurofeedback (NFB) training. The real NFB training induced cortical activation in the somatosensory and motor-related areas (**A1**), while the sham NFB training induced SMA activation (**A2**). (**B**) Relationships between Oxy-Hb gain in the hand area of the left primary motor cortex (lateral Lt-M1) and performance gain in the peg board task (**B1**), and those between cortical activity in the FPA during the performance of the real NFB training on the 6th day of the training, and cortical activity in the somatosensory and motor-related areas during performance of the peg board task after the real NFB training (**B2**). The dotted lines indicate regression lines. The data in each circle indicate data from each subject.

Previous studies suggest that the primary motor area (M1 area) is implicated in skilled motor learning, where functional reorganization and synaptic plasticity occur [12,24]. Since the FPA projects to the M1 area indirectly by way of other cortical areas including the dlPFC [25,26,27], the FPA might reorganize synaptic connections in the M1 areas to improve motor skills. Previous tDCS studies reported that M1 tDCS reorganized not only functional connectivity within the M1 area, but also functional connectivity between the M1 and other cortical areas, and between the M1 and subcortical areas to improve motor rehabilitation [28,29,30]. Therefore, tDCS over the FPA may facilitate the FPA role to improve motor learning, partly through induction of reorganization and synaptic plasticity in the M1 area. Ota et al. (2020) investigated this possibility by activating the FPA by means of neurofeedback (NFB) training instead of tDCS [20]. In NFB training, half of the subjects were shown hemodynamic cortical activity of their own FPA on a monitor (real NFB training), while the remaining subjects were shown randomized false activity (sham NFB training). All subjects received the NFB training for 6 days, in which they performed imagery of a peg board task to increase FPA activity under the feedback of their own FPA or randomized false activity. Before and after the NFB training, the subjects received NIRS studies to assess brain hemodynamic activity during the performance of the peg board task. After the NFB training, the subjects with the real NFB training exhibited hemodynamic cortical responses in the left somatosensory and motor-related areas including the premotor area (PMA), M1 area, and primary somatosensory cortex (S1) (Figure 4(A1)), while the subjects with the sham NFB training exhibited hemodynamic cortical responses in the supplementary motor area (SMA) (Figure 4(A2)). Additional analyses indicated that cortical activity gain in the hand area of the M1 (lateral part of the left M (lateral Lt-M1)), which was defined as activity in the lateral Lt-M1 area before the NFB training divided by that after the NFB training, was significantly linked to performance gain, defined as peg scores before the training divided by those after the training (Figure 4(B1)). Furthermore, cortical activity in the left somatosensory and motor-related areas during the performance of the peg board task after the NFB training was significantly and positively linked to cortical activity in the FPA during the performance of the imagery on the last day of the NFB training (Figure 4(B2)). These results suggest that the FPA reorganizes synaptic connectivity patterns in the M1 area, so that reorganized activity patterns of M1 neurons in the M1 area are more suitable for the peg board task [20]. Interestingly, in the subjects with the sham NFB training, cortical activity in the SMA increased during the performance of the peg board task after the training (see above). The SMA has been proposed to function as “an action monitoring system” that provides warning signals for errors and incorrect responses [31]. In the sham NFB training, the participants were shown randomly generated feedback signals that were irrelevant to performing the peg board task, suggesting that the FPA could lead to erroneous reorganization of the synaptic connectivity patterns in the M1 area during the sham NFB training. These findings suggest the SMA activity after the sham NFB training is involved in detection of erroneous synaptic activity in the somatosensory and motor-related areas, which may promote reformation of correct (new) association between incoming sensory inputs and motor responses.

## 3. Parkinson’s Disease (PD)

### 3.1. Effects of tDCS over the M1, Dorsolateral PFC (dlPFC), and Cerebellum

This section briefly describes the current status of tDCS in PD. The main symptoms in PD include motor symptoms (tremors at rest, rigidity, bradykinesia, freezing of gait, and impaired postural reflexes). PD also exhibits nonmotor symptoms (e.g., cognitive deficits, depression, orthostatic hypotension, REM sleep abnormalities, etc.) that tend to appear before the motor symptoms occur [32]. Anatomically, PD is characterized by gradual depletion and degeneration of dopamine neurons, especially in the midbrain substantia nigra pars compacta (SNc) and ventral tegmental area (VTA), resulting in network dysfunction of cortico-basal ganglia circuits (dysfunction of the direct and indirect pathway system) [33,34,35]. Furthermore, cortical network disruption may be involved in PD symptoms. Tessitore et al. (2012) reported that severity of freezing of gait was associated with a reduction of functional connectivity within the two cortical networks: executive-attention (PFC) and visual (right occipito-temporal gyrus) networks [36]. In addition, executive function, which is mainly supported by the dlPFC, is positively associated with hand dexterity [37,38,39]. These findings suggest that the target brain areas for PD treatment should include not only the cortico-basal ganglia motor circuits, but also cortical circuits such as the dlPFC.

Deep brain stimulation (DBS) is a recognized surgical intervention for the treatment of PD that can inhibit (ameliorate) overactivity of the subthalamic nucleus (STN) or internal segment of globus pallidus (GPi), while regenerative therapy is attracting attention as a state-of-the-art treatment aiming for a radical cure. Nevertheless, these therapies are not readily accessible, and drug therapy remains the most common and essential treatment for PD. Furthermore, long-term use of medication often results in side effects such as wearing-off and dyskinesia. In light of the issues in drug therapy, neuro-rehabilitation using NIBS has recently garnered attention in the context of PD [40,41,42,43,44,45,46].

In clinical studies of tDCS in PD patients, two stimulation sites are often used as regions of interest (ROI): the M1 and dlPFC. The tDCS studies in PD reported that anodal stimulation of the M1 improved motor symptoms [43,44] (Supplementary Table S1), while anodal stimulation of the dlPFC improved executive and cognitive functions [45,46] (Supplementary Table S1). Although the sample size was small, many new reports on PD suggest that tDCS treatment is effective. However, the neural mechanism of improvement is not yet fully clarified, and further accelerated research is necessary. Moreover, it may not be the best treatment to solely stimulate motor-related areas to improve motor function in PD. To develop stimulation sites for tDCS, it is essential to actively use functional MRI and other techniques to assess after-effects of tDCS to induce functional changes in the stimulated areas.

The neural network connecting the cerebellum and the basal ganglia [47] is gaining more clarity, as are the projections from the cerebellum (dentate nucleus) to the PFC and other areas via the thalamus [48]. This has led to increased interest in the relationship between PD and the cerebellum. Furthermore, research suggests that activities in the cerebellum and PFC, including the FPA, are functionally linked in humans [49]. Based on the findings, researchers suggest that applying tDCS to the cerebellum during motor practice could enhance motor skills in PD. de Albuquerque et al. [50,51] investigated the impact of anodal cerebellar tDCS (c-tDCS) on the upper extremity function of PD patients while they learned to perform a complex optokinetic isometric precision grip strength task (PGT). They found that applying c-tDCS to the cerebellum did not result in a significant improvement in motor skill acquisition for hand and arm tasks [50,51]. While there is no current evidence to suggest an improvement in upper limb function, Workman et al. conducted a study on cerebellar functions such as “balance and walking ability”. Their findings showed significantly higher Berg Balance Scale scores with the application of high-intensity cerebellum stimulation (4.0 mA × 20 min) [52]. These findings suggest that c-tDCS may require a higher current intensity due to the cerebellum being covered by a relatively thick occipital bone, or anatomical/physiological properties of the cerebellum different from the cortex.

### 3.2. Effects of tDCS over the FPA in PD Patients

**Figure 5:**
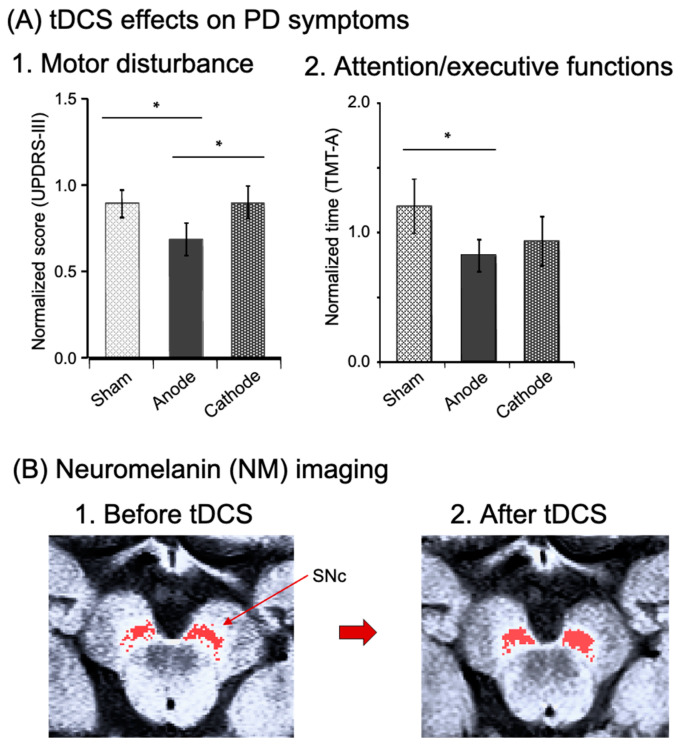
Effects of tDCS over the FPA on PD symptoms (**A**) (reproduced from Ishikuro et al., 2018 [15] under a CC-BY license) and neuromelanin (NM) imaging in the midbrain (**B**) (created from the original NM-MRI data by Ishikuro et al., 2021 [19]). (**A**) Effects of the tDCS on motor disturbance (**A1**) and nonmotor functions (attention/executive functions) (**A2**). Motor disability was evaluated with the Unified PD Rating Scale (UPDRS (part III: motor examination)) after intervention of each tDCS stimulation (anodal, cathodal, and sham tDCS). Nonmotor functions were assessed with the Trail Making Test A (TMT-A). ∗, *p* < 0.05. (**B**) Imaging of dopamine neurons by neuromelanin magnetic resonance imaging (NM-MRI) in the substantia nigra compacta (SNc) before (**B1**) and after (**B2**) FPA tDCS in one PD patient. Red pixels indicate NM-sensitive areas in the SNc, where dopamine neurons are located.

The previous sections suggest that tDCS over the M1 area or dlPFC improves motor or nonmotor symptoms in PD, and that tDCS over the FPA promotes motor learning through reorganization of M1 activity in healthy people, further suggesting that tDCS over the FPA may ameliorate PD symptoms. Ishikuro et al. [18] investigated effects of tDCS (anodal, cathodal, and sham tDCS) over the FPA on motor and nonmotor symptoms in a cross-over design in PD patients [18] (Supplementary Table S1). They reported that anodal tDCS significantly reduced normalized scores of motor disability in the Unified PD Rating Scale (UPDRS (part III)) (Figure 5(A1)), and significantly raised scores of motor functions in the Fugl Meyer Assessment set (FMA), while it significantly reduced time to complete a high dexterity task in STEF [18]. The same anodal tDCS also improved nonmotor function: reduction of normalized time required to complete the Trail Making Test A (TMT-A) to assess attention/executive functions (Figure 5(A2)). Thus, the tDCS of the FPA ameliorated not only motor, but also nonmotor symptoms in PD.

Dopamine neurons in the SNc and VTA receive glutamate transmissions directly or indirectly from the PFC [53,54,55,56]. Additionally, dopamine neuron activity is functionally associated with PFC neuron activity [57,58], and tDCS of the PFC can substantially raise dopamine and tyrosine levels in PD model mice [59]. Therefore, it is plausible to assume that FPA stimulation, which likely sends excitatory projections to midbrain dopamine neurons or activates the dlPFC projecting to dopamine neurons, could impact dopamine cells in the SNc of individuals with PD. It is also possible that tDCS over the FPA could enhance brain regions linked to neuromelanin (refer to below). Neuromelanin is a brown pigment that accumulates in neurons containing catecholamines, and is abundant in dopaminergic cells in the SNc and noradrenergic cells in the locus coeruleus (LC). Recently, fMRI studies reported interesting findings that neuromelanin is closely associated with clinical symptoms of PD assessed using UPDRS-III [60,61]. Neuromelanin can be relatively easily imaged by T1-weighted 3T-MRI [62,63]. Ishikuro et al. (2021) examined effects of tDCS over the FPA in one PD patient as a case report, using noninvasive imaging of neuromelanin to reveal dopamine neurons [19]. They reported that the same tDCS protocol (i.e., 2 weeks of rehabilitation with tDCS in the FPA) increased the neuromelanin-sensitive area in the SNc, where dopamine neurons are located, from 43.2 to 53.2 mm^2^ (increases by 18.8%), and that the patient exhibits clinically meaningful improvement of motor deficits (Figure 5B). The PFC provides direct or indirect excitatory signals with dopamine neurons (as discussed previously). The survival of dopamine neurons or expression of a dopaminergic phenotype is activity-dependent [64,65]: a decline in electrical activity in dopamine neurons results in cellular death or a reduction of tyrosine hydroxylase (which is the rate-limiting enzyme for dopamine synthesis). Consequently, tDCS over the FPA might support the survival of dopamine neurons and promote the expression of tyrosine hydroxylase, possibly resulting in increases in the area sensitive to neuromelanin imaging. Furthermore, anodal tDCS of the frontal cortex for 3 weeks raised dopamine content of the entire brain in a mouse PD model [59]. Since dopamine is crucial for executive functions in the PFC [66,67], these findings indicate that tDCS over the FPA, combined with physical rehabilitation, might cause plastic changes in the dopamine neurons of PD patients, resulting in an improvement of both motor and nonmotor symptoms (e.g., deficits in executive functions).

## 4. Neural Mechanisms of Effects of FPA tDCS

**Figure 6:**
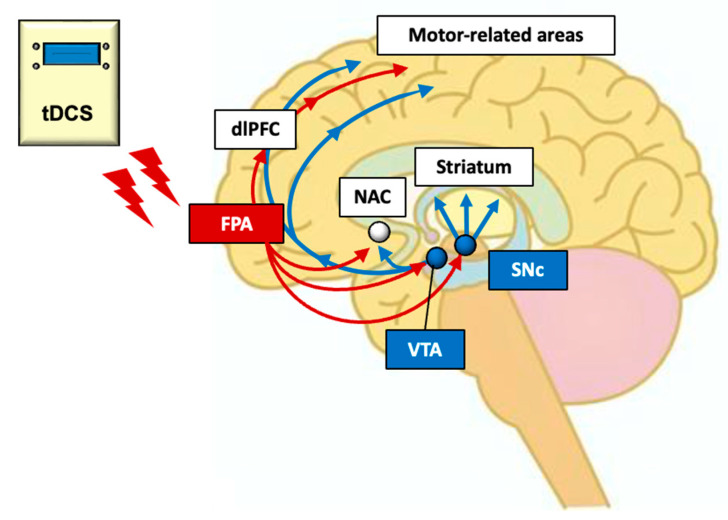
Hypothetical neural mechanisms of tDCS over the FPA in neuro-rehabilitation. Dopamine neurons in the SNc and VTA receive excitatory glutamatergic transmission directly and/or indirectly from the PFC. Thus, tDCS stimulation of FPA, which sends projections to midbrain dopamine neurons, may affect dopamine cells in the SNc of PD patients. Furthermore, tDCS over the FPA might reorganize the functional connectivity of the M1 area through the dlPFC and nucleus accumbens (NAC). Red arrows indicate direct and/or indirect projections from the FPA, while blue arrows indicate dopamine projections.

The previous sections indicate that anodal tDCS of the FPA, which raises the excitability of pyramidal neurons in the FPA, may facilitate or improve motor and nonmotor functions in healthy subjects and PD patients. Multiple mechanisms may be responsible for these effects (Figure 6). First, tDCS over the FPA may reorganize the functional connectivity of the M1 area to improve motor learning, as shown in the study with NFB to activate the FPA. The FPA projects to the dlPFC [26], which controls the excitability of the ipsilateral M1 area [25]. Furthermore, the FPA and M1 area are functionally connected [68]. These findings suggest that tDCS over the FPA might reorganize the functional connectivity of the M1 area through activity-dependent synaptic plasticity (e.g., LTP). Second, tDCS over the FPA might facilitate nonmotor cognitive functions (e.g., executive function) through FPA projections to the dlPFC. Consistently, a meta-analysis study reported that the FPA coactivates with the dlPFC (areas 9/46) in various tasks including working memory tasks [69]. Furthermore, the lateral FPA is believed to have a crucial role in executive function and stimulus-oriented action [70,71]. Third, tDCS over the FPA might exert its effects by acting on dopamine neurons. Midbrain dopamine neurons receive direct and/or indirect glutamatergic excitatory afferents from the PFC [53,54,55,56], and activity of dopamine neurons correlates to that of PFC neurons [57,58], suggesting that elevated FPA activity by tDCS may promote dopamine release in the somatosensory and motor-related areas, as well as the basal ganglia. Since dopamine facilitates LTP induction as well as motor skill learning [72,73,74], increased dopamine release in the somatosensory and motor-related areas may facilitate the first mechanism in synaptic plasticity. In the case of PD, increases in NM-sensitive areas, where dopaminergic neurons are located, by tDCS may increase dopamine release in the cortico-basal ganglia circuits as well as in the PFC, which contributes to the amelioration of PD symptoms (see above section). Fourth, elevated FPA activity by tDCS could enhance one’s motivational drive and facilitate the process of motor learning. Hosoda et al. [75] showed the crucial role of the FPA in persistence during difficult motor learning, whereas Soutschek et al. [76] found that tDCS over the FPA enhances motivation for cognitive and physical efforts. These motivational changes associated with the FPA are critical in physical rehabilitation, since a significant number of patients drop out of rehabilitation programs [77,78,79], and patients with PD have difficulties with mental persistence [80]. The role of the FPA in persistence may be attributed to its projection to the nucleus accumbens. Previous neuropsychological studies reported that deficits in motivation are associated with deficits in goal-directed behaviors in depression [81], reminiscent of dropping out of physical rehabilitation, and functional connectivity between the FPA and nucleus accumbens is more decreased in patients with more severe symptoms in depression [82], while dopamine depletion in nucleus accumbens decreased the preference for a high-effort option with high reward [83,84]. Furthermore, manual dexterity was disturbed in patients with depression [85], while activity of nucleus accumbens was increased during a manual task in subjects with spinal lesions compared with intact subjects. Additionally, functional connectivity between the nucleus accumbens and M1 area was increased during relearning of a hand motor task after spinal lesions in monkeys [86]. The findings suggest that the neural circuits comprising the FPA, nucleus accumbens, and dopaminergic neurons may promote both effortful learning of manual motor tasks and preservation of meticulous manual dexterity. In summary, the neural mechanisms impacted by tDCS over the FPA in motor learning are complex and involve multiple processes that contribute to improvement of fine manual dexterity, which may facilitate rehabilitation.

## 5. Future Issues on tDCS over the FPA

We have reviewed the efficacy of tDCS over the FPA, which suggests that this technique might be useful in clinical applications, not only in healthy subjects, but also in PD. However, upcoming challenges pertaining to tDCS over the FPA need to be addressed. First, optimal conditions for tDCS over the FPA (current intensity, duration, location, etc.) should be determined. Inconsistency of clinical efficacy of tDCS among studies may be ascribed to different stimulation protocols [87]. Recently, high-definition tDCS (HD-tDCS) has been introduced, which allows more focused current delivery [88]. HD-tDCS should be applied to the FPA for more focused stimulation to better understand roles of the FPA in rehabilitation. Second, only a small sample of patients with PD were tested with tDCS over the FPA in the previous studies [15,16]. Further studies in larger populations are needed to determine clinical efficacy of tDCS over the FPA. Third, although applying FPA tDCS improved both motor and nonmotor functions of PD patients, further studies are needed to clarify the time-lapse of improvements during repeated interventions, as well as the time-lapse of changes in those improvements after repeated interventions. Fourth, there are multiple forms of motor learning including use-dependent motor learning (repetitive motor learning), instructive motor learning (strategy-based motor learning), reinforcement motor learning, and sensorimotor adaptation-based motor learning [89]. Although repetitive motor learning is supposed to play a major role in rehabilitation, these multiple forms of motor learning could occur independently and simultaneously [89]. Furthermore, these multiple forms of motor learning could interact with each other [90]. Further research is needed to clarify how the tDCS over the FPA modulates each form of motor learning, and how it modulates the interactions between various learning mechanisms during physical rehabilitation. 

## 6. Conclusions

Emerging evidence suggests that tDCS can safely modulate neuronal excitability by producing either somatic depolarization (anodal stimulation) or somatic hyperpolarization (cathodal stimulation), based on the polarity of a weak current from the scalp. This technology has become increasingly popular in the field of rehabilitation medicine for treating neurodegenerative illnesses, such as PD.

Previous studies have primarily applied tDCS to the M1 region in PD patients, and have reported the usefulness of tDCS in neuro-rehabilitation. Recent neuropsychological and clinical studies have reported that tDCS of the FPA can improve motor learning and motor functions in both healthy participants and patients with PD. tDCS over the FPA may promote motor skill learning through its effects on the M1 area and/or midbrain dopamine neurons. Furthermore, recent studies have revealed additional distinctive effects of tDCS of the FPA, including impacts on persistence and motivation, which are important for rehabilitation. These results suggest that the FPA could be a new target for the application of tDCS in neuro-rehabilitation.

## Data Availability

The data presented in this study are available on request to the corresponding author. The data are not publicly available due to privacy of the patients.

## Supplementary Materials

The following supporting information can be downloaded at: https://www.mdpi.com/article/10.3390/brainsci13111604/s1, Table S1: Summary of tDCS studies in patients with PD.
