# Supplementary material for: Neural Mechanisms of Neuro-Rehabilitation Using Transcranial Direct Current Stimulation (tDCS) over the Front-Polar Area
Source: Brain Sci. 2023 Nov 18;13(11):1604. doi: 10.3390/brainsci13111604 (PMC10670271; doi:10.3390/brainsci13111604)
Supplement: Supplementary file 1 [file brainsci-13-01604-s001.zip › brainsci-2672382-supplementary.pdf]

Neural Mechanisms of Neuro-Rehabilitation Using Transcranial Direct Current Stimulation (tDCS) over the Front-polar Area

Ishikuro et al.

Supplementary information

Supplementary Table S1: see the next page.

Supplementary Table S1. Summary of tDCS studies in patients with PD.

Author	Causes of PD	Subjects	Age (years)	Disease duration (years)	Disease progression<br>(Hoehn & Yahr (H&Y) / UPDRS)	Drug	Experimetal design	tDCS (stimulation condition)	Anode	Cathode	Outcome	Results
Kaski et al. (2014)	Ideopathic	16<br>(physical training with/without tDCS, 8; no physical training with/without tDCS, 8)	NA	NA	NA	Under constant medication, the assessments were carried out ar the same time of the day to avoid diurnal variation and on/off medication effects.	Double-blind design, 2 groups (cross-over between G1 and G2; cross-over between G3 and G4)<br>・G1: Physical traing+active tDCS<br>・G2: Physical traing+sham tDCS<br>・G3: No physical traing+active tDCS<br>・G4: No physical traing+sham tDCS	<Active tDCS><br>Current：2.0 mA<br>Duration：900 sec<br>Electro size: anode, 40 cm^2; cathode, 16 cm^2<br>Session：NA<br><Sham tDCS><br>NA	Primary and premotor cortex (a region 10-20% anterior to Cz)	Inion	1) Gait velocity<br>2) Stride length<br>3) TUG<br>4) 6-minute walk test<br>5) Pull	Relative changes after the each intervention were compared.<br>1) Gait velocity: relative change (%) was greater for tDCS + physical training than physical training alone.<br>2) Stride length: relative change (%) was greater for tDCS + physical training than physical training alone.<br>3) TUG: relative change (%) was greater for tDCS + physical training than tDCS alone.<br>4) 6-minute walk test: relative change (%) was greater for tDCS + physical training than physical training alone.<br>5) Pull: relative change (%) was greater for tDCS + physical training than tDCS alone.
Fregni et al. (2006)	Ideopathic	17 (Male, 11; Female, 6)<br>Exp.1a: Anodal tDCS over M1: 9 (Male, 5; Female, 4)]<br>Exp.1b: Cathodal tDCS over M1: 8 (Male, 7; Female, 1)<br>Exp.1c: Anodal tDCS over DLPC: 9 (same subjects as those in Exp.1a)<br>Exp.2: the same subjects as those in. Exp. 1a and 1b.	62.3 ± 1.6<br>(Exp.1a, 1c, 2: 59.2 ± 3.3; Exp.1b, 2: 65.9 ± 4.6)	12.3 ± 1.6<br>(Exp.1a, 1c, 2: 13.7 ± 2.7; Exp.1b, 2: 10.7 ± 1.7)	H&Y: 2.4 ± 0.2<br>(Exp.1a, 1c, 2: 2.4 ± 0.2; Exp.1b, 2: 2.3 ± 0.3)<br>UPDRS(III): 37.7 ± 3.9<br>(Exp.1a, 1c, 2: 36.9 ± 5.0; Exp.1b, 2: 38.2 ± 4.4)	Levodopa: 615 ± 63.1 mg<br>(Exp.1a, 1c, 2: 681.2 ± 67.1 mg; Exp.1b, 2: 539.2 ± 110.9 mg)	Double-blind design, 3 interventions (Exp. 1a, Exp. 1b, Exp. 1c).<br>Both active and sham tDCS were tested in each Exp.<br>Interval between the interventions (active vs. sham) in each experiment: at least 48 hours	<Active tDCS><br>Current：1.0 mA<br>Duration：1200 sec<br>Electro size：35 cm2<br>Session：1<br><Sham tDCS><br>Duration: 5 sec<br>The other conditions were the same as those for Active tDCS.	Exp. 1a: anode, primary motor cortex (M1); cathode, contralateral supraorbital area<br>Exp. 1b: anode, contralateral supraorbital area; cathode, primary motor cortex<br>Exp. 1c: anode, DLPFC; cathode, contralateral supraorbital area		<Exp.1><br>1) sRT, 2) UPDRS-III, 3) PPT<br><Exp.2><br>MEP from the right FDI	Relative changes (%) from the base line in active tDCS were compared with those in sham tDCS in Exp.1a and 1b. In Exp.1c, relative changes by three interventions (anodal M1 stim. vs. sham M1 stim. vs. anodal DLPFC stim.) were compared.<br><Exp. 1a (anodal tDCS over M1)><br>1) sRT: significant improvement by active tDCS<br>2) UPDRS-III: significant improvement by active tDCS<br>3) PPT: ns<br><Exp. 1b (cathodal tDCS over M1)><br>1) sRT: ns<br>2) UPDRS-III: ns<br>3) PPT: ns<br><Exp. 1c><br>1) sRT: significant differences between anodal M1 stim. and sham M1 stim, but no significant differences between anodal DLPFC stim. and sham M1 stim.<br>2)UPDRS-III: same results as for sRT.<br><Exp.2><br>MEP amplitude: significant increases by anodal M1 stim. compared with sham M1 stim., significant decreases by cathodal M1 stim. compared with sham M1 stim.
Benninger et al. (2010)	NA	25<br>Sham: Male, 7; Female, 5<br>Active: Male, 8; Female, 4	Sham: 64.2 ± 8.8<br>Active: 63.6 ± 9.0	Sham: 9.1 ± 3.3<br>Active: 10.6 ± 7.1	H &Y(ON state)<br>Sham: 2.4 ± 0.2<br>Active: 2.5 ± 0.1<br>H &Y(OFF state)<br>Sham: 2.9 ± 0.4<br>Active: 2.7 ± 0.3	Levodopa equivalent daily dose (LEDD) (mg/day)<br>Sham: 1287.7 ± 808.8<br>Active: 1024 ± 541.5	Randomized, double blind, sham-controlled study	<Active tDCS><br>Current：2.0 mA<br>Duration：1200 sec<br>Electro size：anode, 97.5 cm^2; cathode, 25 cm^2<br>Session：8 sessions<br><Sham tDCS><br>Current：1.0 mA<br>Duration：60 - 120 sec<br>Electro size：9 cm^2<br>Session：8 sessions	<Active tDCS><br>Pre-and motor area (10 mm anterior to Cz) or prefrontal cortices (forehead above eyebrows)<br><Sham tDCS><br>Forehesd	<Active tDCS><br>Mastoids<br><Sham tDCS><br>Forehesd (cathode was placed 1 cm apart from anode)	1) Gait: time to walk 10 meters<br>2) Bradykinesia: hand-closing and opening, elbow-flexion, hand-closing and opening, elbow-extension<br>3) UPDRS (ON/OFF states)<br>4) Serial reaction time task<br>5) Other assessments (BDI, SF-12v2, self assessment)	1) Gait: significant decreases in active tDCS compared with sham tDCS 1 day after the intervention in OFF state.<br>2) Bradykinesia: significant decreases of movement time in active tDCS compared with sham tDCS 1 day, 1 month, and 3 months after the intervention in both ON and OFF states.<br>3) UPDRS: significant decreases of composite UPDRS bradykinesia scores in active tDCS compared with sham tDCS 1 day after the intervention in OFF state.<br>4) Serial reaction time task: ns
Boggio et al. (2006)	Ideopathic	18 (Male, 12; Female, 6)<br>(Exp.1, 9; Exp.2, 9)	61.1 (45 - 71)<br>Exp.1: 59.2 ± 9.9<br>Exp.2: 61.0 ± 12.1	Exp.1: 13.7 ± 8.2<br>Exp.2: 12.7 ± 8.1	<Exp.1><br>H&Y: 2.3 ± 0.9<br>UPDRS: 36.8 ± 18.5<br>MMSE: 24.4 ± 3.1<br><Exp.2><br>H&Y: 2.4 ± 0.7<br>UPDRS: 43.0 ±13.7<br>MMSE: 24.9 ± 3.5	Wthdrawal of antiparkinsonian drugs for more than 12 hours	3 conditions (anodal M1 stim. vs. anodal DLPFC stim vs. sham), cross-over study in each Exp.<br>Interval between the conditions：48 hours	<active tDCS in Exp.1><br>Current: 1.0 mA<br>Duration：1200 sec<br>Electro size：35 cm^2<br>Session：1 session<br><active tDCS in Exp.2><br>Current: 2.0 mA<br>The other condtions were the same as those in Exp.1.<br><sham tDCS in Exp.1 and 2><br>Current intensity was gradually decreased during initial 30 sec.	<Active tDCS><br>Primary moptor cortex (M1)<br>or<br>Left dorsolateral prefrontal cortex (L-DLPFC)<br><Sham tDCS><br>L-DLPFC	<Active and sham tDCS><br>Contralateral right orbit	Working memory (three-back letter working memory paradigm)<br>The task was performed two times in each condition of each Exp.: 5 min before the tDCS and during the last 5 min of each tDCS.	Exp.1:<br>ns among the 3 condituons in 3 parameters (correct responses, errors, and reaction time).<br><br>Exp.2：<br>1) significant increases of correct responses during L-DLPFC compared with M1 and sham tDCS<br>2) significant decreases of errors during L-DLPFC tDCS compared with sham tDCS.<br>3) ns in reaction time among the 3 conditions
Doruk et al. (2014)	Ideopathic	18 (Male, 12; Female, 6): L-DLPFC group, 6; R-DLPFC group, 5; sham group, 7.	61 ± 8 (40 - 71)	NA	NA<br>(the patients showed at least two out of three cardinal motor features of PD, and a sustained and significant response to dopaminergic treatment)	Stable medication at least 30 days prior to the enrollment	Comparative study of 3 groups	<Active tDCS><br>Current：2.0 mA<br>Duration：1200 sec<br>Electorode size：35 cm^2<br>Session：10 times over 2 weeks (Monday-to-Friday)<br><Sham tDCS><br>Current was delivered only during the initial 30 sec.	Group 1: L-DLPFC (F3)<br>Group 2: R-DLPFC (F4)<br>Group 3: sham (L or R-DLPFC)	Group 1: the right supraorbital region<br>Group 2: the left supraorbital region<br>Group 3: sham (L or R-DLPFC)	Following assessments were performed 3 times: before intervention, after intervention, and 1 month after intervention.<br>1) Cognitive Assessment [TMT (A&B), WCST, PCL, WM using the forward and backward Digit Span Tests and 3-back Test, stroop test, the Hooper Visual Organization Test (HPVOT), CPM, MMSE]<br>2) Behavioural Assessment [BDI, HRSD, HAS]<br>3) Motor assessment [UPDRS-III, SRT, 4-CRT, PPT, FT, WT, BU&SP)	1) TMT(B):<br>significant improvement in the L-DLPFC and R-DLPFC groups compared with sham group 1 month after the experiment<br>2) BDI：<br>Significant reduction of BDI scores in the L-DLPFC group compared with the sham group.<br>3) All motor assessments: ns
Ishikuro et al. (2018)	Ideopathic	9 (Male, 3: Female, 6)	77.5 ± 4.8 (68 - 83)	69.2 ± 30.7 (11 - 108)	Yahr: 1 - 3	During the study, no phamacological medication was provided	3 conditions (Anode vs. Cathode vs. Sham), cross-over study<br>Interval between the groups：36 hours	<Active tDCS><br>Current：1.0 mA<br>Duration：900 sec<br>Electro size：35 cm^2<br>Session：5 times<br><Sham tDCS><br>Current was delivered only during the initial 30 sec.	Front-polar area (FPA)	Occipital area (OPA)	1) UPDRS-III<br>2) Fugl-Mayer Assessmen (FMA)<br>3) Simple Test for Evaluating hand<br>4) Function (STEF)<br>5) Trail making test (TMT) (A)	Significant improvement in Anode condition compared with Sham or Cathode condition in the following batteries (normalized score: relative scores compared to pre-intervention scores)<br>1)UPDRS-III: 0.69 ± 0.15<br>2) FMA: 1.06 ± 0.06<br>3) STEF: 1.12 ± 0.13<br>4) TMT-A: 0.82 ± 0.12

NA, not available; sRT or SRT, Simple reaction time; UPDRS, unified parkinson's desease rating scale; FDI, the first dorsal interosseous; PPT, Purdue Peg-board test; BDI, beck depression inventry; TMT, trail maiking test; WCST, Wisconsin Card Sorting Test; PCL, Probabilistic Classification Learning; WM, Working Memory Test; HPVOT, the Hooper Visual Organization Test; CPM, Colored Progressive Matrices; MMSE, Mini Mental Status Examination; HRSD, the Hamilton Rating Scale for Depression; HAS, the Mamilton Anxiety Scale; 4-CRT, 4-Choice Reaction Time; FT, Finger Tapping; WT, Walking Time; BU&SP, Buttoning-Up and Supination-Pronation
